# Adverse Drug Reaction Reporting in Ethiopia: Systematic Review

**DOI:** 10.1155/2020/8569314

**Published:** 2020-08-10

**Authors:** Abel Demerew Hailu, Solomon Ahmed Mohammed

**Affiliations:** ^1^Department of Pharmacy, Dessie Health Science Collage, Dessie, Ethiopia; ^2^Department of Pharmacy, College of Medicine and Health Science, Wollo University, Dessie, Ethiopia

## Abstract

Adverse drug reactions are major global public health problems and an important cause of mortality. Problems related to medicines safety can emerge from real-life medication use due to increasing access to complex treatment of concomitant infectious and noncommunicable diseases, hence leading to a higher prevalence of drug-related problems. The objective of this review was to assess the knowledge, attitude, and practice of adverse drug reaction reporting among health care professionals in Ethiopia. Relevant literatures were searched from Google Scholar, PubMed, Hinari, Web of Science, Scopus, and Science Direct using inclusion and exclusion criteria. From 133 searched studies, 13 studies were reviewed. The knowledge and attitude of health care professionals towards adverse drug reaction reporting ranged from 22.68% -60.33% and 47.22% -67.14%, with averages of 41.50% and 57.18%, respectively. While 46.93% encountered adverse drug reactions and 41.8% reported in the last 12 months. One-third (34.15%) of health care professionals do not know how to report adverse drug reactions. Fearing to report, uncertainty about the adverse drug reaction, concern about reporting generating extra work, thinking that one report does not make any difference, nonavailability of reporting forms, and lack of feedback from regulatory authority were the stated reasons for underreporting. We conclude that the knowledge, attitude, and practice of health care professionals towards spontaneous ADR reporting were low. Conducting awareness and educational training and implementation of electronic reporting can improve the ADR reporting practice.

## 1. Introduction

An adverse drug reaction (ADR) [1] is a response to a drug that is noxious, unintended, and which occurs at doses normally used in humans for prophylaxis, diagnosis, or therapy of disease or the modification of physiologic function [2]. Quality, efficacy, and safety assured medicines are essential in clinical practice [3]. Problems related to medicine safety can emerge from real-life medication use. Postmarketing monitoring is, therefore, an important step to detecting medicine-related problems that were not possible to identify during the pre-marketing phases [4].

ADR are major global public health problems [5] and an important cause of mortality [6]. In some countries, ADR rank among the top 10 leading causes of mortality [7]. Globally, the rate of fatal ADR in patients presenting to a hospital has been reported to range from 0.1% to 10% [8]. Studies conducted in developed countries reported that the rate of fatal ADR ranged from 0.05% to 3% of all patients admitted due to an ADR [9, 10]. A recent review of studies also found that the median proportion of ADR resulting in mortality in developing and developed countries was 1.8% and 1.7%, respectively [11].

In Ethiopia, there is increasing access to complex treatment of concomitant infectious and non-communicable diseases, leading to a higher prevalence of drug-related problems [12] due to medication errors, product quality defects, and irrational use of medicines among patients on chronic follow-up in ambulatory care clinics [13] and substantial causes of mortality rate among patients presenting to emergency departments in Ethiopia [14]. Thus, ADR monitoring is one of the main priority agendas of the government of Ethiopia because the prevention of ADR helps to minimize the consequential undesirable effects [15].

Healthcare professionals are obliged to be vigilant in detecting and reporting suspected ADR to the Ethiopian Food and Drug Administration (EFDA). This will help the administration to take action to prevent or minimize the occurrence of medicine-related injuries [16]. Besides, patients who suspect and experience adverse drug events are expected to report to health care professionals and the national medicine regulatory administration [17].

The Uppsala Monitoring Center is the international database of ADR reports and currently about 4.7 million case reports received from several national centers. However, still, it is estimated that only 6-10% of all ADR are reported. Although Ethiopia is participating in the program, its contribution to the Uppsala Monitoring Center database is very small. This is essentially due to the absence of a vibrant ADR monitoring system and also a lack of a reporting culture among healthcare workers [18, 19].

Spontaneous reporting has contributed significantly to successful pharmacovigilance. The contribution of health professionals, in this regard, to ADR databases, is enormously significant and has encouraged ongoing ascertainment of the benefit-risk ratio of some drugs as well as contributing to signal detection of unsuspected and unusual ADR previously undetected during the initial evaluation of a drug. In Ethiopia, voluntary reporting has been effective as in 2010. Despite rigorous activities performed by the EFDA, the level of awareness of health care providers towards ADR reporting was not satisfactory [20]. Evaluating their knowledge, attitude, and practice can help in devising strategies to improve reporting schemes. Hence, this review was intended to review the knowledge, attitude, and practice of health care professionals toward ADR reporting.

## 2. Materials and Methods

### 2.1. Search Strategy

A systematic literature search was conducted in Google Scholar, PubMed, Hinari, Web of Science, Scopus, and Science Direct electronic databases for articles published between January 2000 and July 2020. Some studies were also identified through a manual Google search and the reference lists of retrieved articles. The entire searches were done from July 23 to 25, 2020, using key words “Adverse drug reaction”, “ADR”, “Knowledge”, “Attitude”, “Practice”, and in combination.

### 2.2. Article Selection

Studies were included in the review if they aimed to assess the knowledge, attitude, and practice of health care professionals toward ADR reporting. Studies that were written in English, open access in portable document formats, and all study designs were included, while those studies published only as dissertations, abstracts, editorials, or clinical opinion, and published before 2000 were excluded.

### 2.3. Assessment of Methodological Quality

Methodological quality assessment was done prior to inclusion of selected articles to ensure that the data extraction met the quality criteria using preferred reporting items for systematic reviews and meta-analysis (PRISMA) flow diagram and guidance set out by the center for reviews and dissemination [21]. Each of the 13 studies was evaluated for each criterion/question and rated it as “Yes” with score 1; if described partly, we scored it as 0.5, then 0 for “No.” Then, the total score was calculated by summing each score and score less than 75% graded as low quality, 75% to 90% graded to moderate quality, and greater than 90% was graded as high quality. Two reviewers were involved in this review and appraised the full text of each study independently. Any discrepancies were resolved through discussion.

### 2.4. Data Abstraction

The author, year, study design, sample size, response rate, town, knowledge, attitude, practices, and perceived factors of ADR reporting were extracted from each study using an abstraction form.

## 3. Result

### 3.1. Literature Search Results

**Figure 1:**
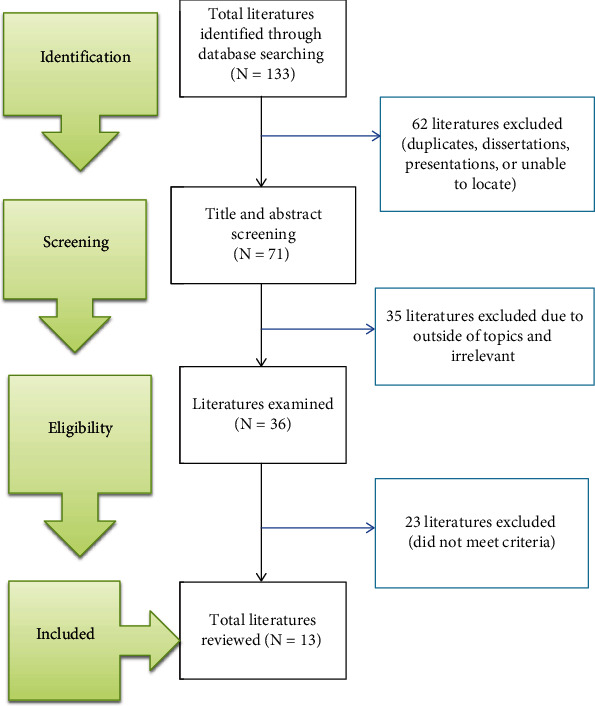
Flow diagram of study selection.

The initial advanced search in all databases yielded 133 studies. Finally, after excluding duplicates and irrelevant studies, 13 studies were reviewed. The figure below briefly describes the flow of the study selection employed (Figure 1).

### 3.2. Methodological Quality of the Included Studies

The reporting quality results revealed that most studies were of high quality (*n* = 10, 76.93%), whereas two (15.38%) were of moderate quality and one (7.69%) were of low quality.

### 3.3. Studies Characteristics

All included studies (13) varied in sample size and location. Response rate and the sample size ranged from 76.1% to 100% and from 82 to 422, respectively. From these articles, all studies were cross-sectional, and nearly half were conducted in health institutions (Table 1).

### 3.4. Knowledge and Attitudes of Health Care Professionals regarding ADR Reporting

From 13 reviewed studies, one study [31] and two studies [23, 25] did not report the knowledge and attitudes of health care professionals towards ADR reporting, respectively. The knowledge and attitude of health care professionals towards ADR reporting ranged from 22.68% to 60.33% and 47.22% to 67.14%, respectively, with an average of 41.50% and 57.18% (Table 2).

Except for one, all studies used a structured self-administered questionnaire to assess the knowledge of health care professionals related to ADR reporting. On average, 45.91% knew the national ADR reporting system, 40.68% knew the availability of ADR reporting form, and 41.59% thought that ADR were the same as with side effects. Health professionals who knew the term pharmacovigilance ranged from 19.51% to 46.4% (Table 3).

The attitude of ADR reporting among health professionals was assessed by administering a set of questions on a scale. ADR should be reported spontaneously at regular base by 78.71%, and reporting ADR is part of the duty of health professionals 83.77% of health professionals. In seven studies, 90.36 respondents agreed on the importance of reporting drug safety for the health care system. Three studies reported that 24.79% of health care professionals agreed that reporting of ADR affects patient's confidentiality issues and fear of legal liability affects ADR reporting 46.94% (Table 4).

### 3.5. Practice of Healthcare Professionals regarding ADR Reporting

The reviewed studies measured health care professionals' practices towards ADR reporting practices by identifying whether they encountered, documented, and reported the ADR or not. Twelve studies revealed the average response of health care professionals and reported that 46.93% encountered patients with ADR in clinical practice, and 41.8% of ADR reported in the last 12 months. One-third (34.15%) of health care professionals do not know how to report ADR. Eight studies showed that 45.76% of health care professionals encountered ADR on the patient's clinical record (Table 5).

### 3.6. Perceived Factors for Underreporting ADR

Only four studies assessed the perceived factors of health care professionals towards ADR reporting. The stated factors for underreporting of ADR include fear to reporting, uncertainty about the drug causing the ADR, concern about the report will generate extra work, thinking that one report does not make any difference, reporting forms are not available when needed, and lack of feedback from regulatory authority (Table 6).

## 4. Discussion

Several numbers of drugs are being come into the market every day. However, their safety remains to be a major concern for patients. In developing and developed countries, the median proportion of ADR-related mortality was 1.8% and 1.7%, respectively [11]. Meta-analysis in the United States revealed that ADR alone excluding medication errors killed over 100,000 people in 1994 and were the fourth to sixth leading cause of death [35]. In the area of pharmaceutical care, ADR monitoring mainly focuses on the detection, management, and reporting of ADR of drugs that may result from drugs that are taken in the normal dose for prevention, prophylaxis, or treatment [36]. Studies have revealed that health professionals should practice ADR reporting as it can save the lives of their patients [37].

The knowledge and attitude of health care professionals towards ADR were 41.50% and 57.18%, respectively. The study conducted by Vallano et al. (2005) reported that lack of knowledge and giving less value for the importance of ADR reporting were obstacles for ADR reporting [38]. A systematic review and meta-analysis in India reported that more than 40% had inadequate knowledge and attitude [39]. Similarly, doctors' knowledge and attitudes were found poor [40]. This high gap might arise from a low level of knowledge of healthcare professionals in their working environment, increasing access to complex treatment [12], medication errors, product quality defects, and irrational use of medicines [13], leading to a higher prevalence of drug-related problems.

In this review, 45.91% of health professionals knew the national ADR reporting system. A large degree of variability between ADE reporting systems was found in a similar review [41]. In India, 55.6% of healthcare professionals were not aware of the existence of the pharmacovigilance program [39]. A simple ADR reporting form and system should be developed and made available throughout all health facilities.

Side effects are minor effects of drugs associated with its pharmacological properties [42]. However, 41.59% think that ADR are the same as with side effects. This may result in excessive healthcare costs through increased mortality, morbidity, and hospital admissions. Therefore, there is an urgent need to upgrade the knowledge of health professionals on different forms of unintended effects of drugs. This is helpful to health professionals to differential things to be reported from minor side effects and different complications arising from ADR.

Although 46.93% of health professionals encountered patients with ADR in clinical practice, 41.8% were reported in the last 12 months. This finding was in line with similar systematic reviews where the practices were generally poor [40], more than half [39], and 74.5% never reported any ADR to pharmacovigilance centers [39]. This showed that most of the healthcare professionals who recognized ADR did not report to the concerned body. Even though ADR reporting is the duty of health professionals and important to the general public, inadequate knowledge, attitude, unavailability of reporting, low level of motivation, and salary of healthcare professionals make them negligent to their work.

Most of the pharmacovigilance systems around the world depend on spontaneous reporting of ADR, where reports are submitted on a voluntary basis from health care professionals [38]. Underreporting of ADR by health care professionals is common in most countries. It has also remained to be a major challenge in Ethiopia. Globally, only 6–10% of all ADR have been reported [1], and there was no significant difference in the median underreporting rates for general practice and hospital-based studies [43]. This revealed that spontaneous reporting was very low. Thus, a high rate of underreporting can delay signal detection and consequently compromise the health of patients [1].

Various studies have identified that the absence of getting feedbacks from the national pharmacovigilance center, inadequate knowledge, lack of awareness about reporting of ADR cases, weak system for reporting, uncertainty on how to report, negligence, fear of legal liability, fear to reporting, lack of time, and difficulty in accessing reporting forms were the main driving factors for underreporting ADR [6, 44, 45]. The identified barriers for underreporting were in line with this review, and educational and awareness-raising programs need to be delivered to healthcare professionals by concerned bodies to improve ADR reporting.

Adverse drug reactions pose major public health, financial, and economic implications. The financial burden of ADR worsens substantially when ADR either cause or extend hospitalization [36]. Since monitoring of ADR is an integral component of patient care, all health care professionals in Ethiopia need to be alert to suspected ADR and make immediate and appropriate actions [6].

The use of electronic ADR reporting along with manual methods of ADR reporting can improve efficiency and accuracy for detecting ADRs [46]. It would also be useful to develop systems to assist healthcare professionals in completing ADR reporting within electronic health records [47]. Moreover, drug regulatory authorities, pharmaceutical companies, and academia should be proactive in the detection, documentation, and reporting of ADR [45].

Inadequate knowledge, attitude, and underreporting of ADR is a bottleneck for modern healthcare delivery, as it makes it difficult to know the accurate prevalence of ADR. Well-functioning pharmacovigilance systems allow identifying, detection, and generation of signals. This review will help stakeholders' efforts for plan and implement strategies related to ADR reporting. As limitations, it is not surprising that the reviewed studies followed a range of nonstandardized data collection tools. Hence, the heterogeneity among studies made compression difficult. The definition of ADRs was also not clearly articulated in the original studies.

## 5. Conclusions

The knowledge and attitude of healthcare professionals toward spontaneous ADR reporting were low. There was also underreporting of ADR by healthcare professionals. Conducting awareness and educational training can fill the observed gap in knowledge and attitude. Simplification of the ADR reporting process, improving access to ADR reporting form, and implementation of electronic reporting combined with other methods for ADR reporting can improve the efficiency of the ADR reporting practice.

## Figures and Tables

**Table 1:** Study characteristics (*n* = 13).

S.no	Authors	Sample size	Sampling method	Study design	Response rate	Year	Study area
1	Angamo et al. [22]	82	Convenience sampling	A cross-sectional study	100%	2012	Jimma
2	Bule et al. [23]	130	Purposive sampling	A prospective cross-sectional	100%	2016	Adama
3	Nadew et al. [24]	422	Simple random sampling	An institution based cross-sectional mixed-methods study design	96%	2020	Addis Ababa
4	Adimasu et al. [25]	214	Stratified proportional random sampling	Hospital based cross-sectional study design	100%	2014	Gondar
5	Kefale et al. [26]	280	Stratified and systematic random sampling	A cross-sectional study	76.1%	2017	Addis Ababa
6	Gurmesa et al. [27]	133	Stratified random sampling	Descriptive cross-sectional study design	100%	2016	Nekemte
7	Kasa et al. [28]	120	Convenience sampling	A cross-sectional study design	95%	2019	Kemisse and Ataye
8	Goshime et al. [29]	422	Simple random sampling	A descriptive cross-sectional study	87.9%	2015	Addis Ababa
9	Denekew et al. [30]	251	Stratified and systematic random sampling	A facility based cross-sectional study design	93.22%	2014	Addis Ababa
10	Gidey et al. [31]	345	Stratified random sampling	An institutional-based cross-sectional study	84.8%	2020	Mekele
11	Kassa et al. [32]	67	Purposive sampling	A cross-sectional study	92%	2017	Dessie
12	Hailua et al. [33]	156	Convenience sampling	A prospective cross-sectional study	96.1%	2014	Gondar
13	Shanko et al. [34]	327	Purposive sampling	A hospital-based descriptive cross-sectional study	91.4%	2017	Harar

**Table 2:** Knowledge and attitudes of health care professionals towards ADR reporting (*n* = 13).

S.no	Authors	Knowledge (%)	Attitude (%)
1	Angamo et al. [22]	22.68	47.22
2	Bule et al. [23]	33.08	67.14
3	Nadew et al. [24]	45.57	54.1
4	Adimasu et al. [25]	23.72	NA^∗^
5	Kefale et al. [26]	38.01	62.87
6	Gurmesa et al. [27]	34.1	NA^∗^
7	Kasa Alemu et al. [28]	32.28	57.69
8	Goshime et al. [29]	52.06	63.32
9	Denekew et al. [30]	49.32	48.1
10	Gidey et al. [31]	34.84	51.26
11	Kassa et al. [32]	50.18	61.38
12	Hailua et al. [33]	NA^∗^	65.56
13	Shanko et al. [34]	60.33	55.87

^∗^NA: not assessed.

**Table 3:** Knowledge of healthcare professionals on ADR reporting (*n* = 13).

Sr.no	Description	Angamo et al. [20] *N* (%)	Bule et al. [21] *N* (%)	Nadew et al. [22] *N* (%)	Adimasu et al. [23] *N* (%)	Kefale et al. [24] *N* (%)	Gurmesa et al. [25] *N* (%)	Kasa et al. Alemu [26]	Goshime et al. [27] *N* (%)	Denekew et al. [28] *N* (%)	Gidey et al. [29] *N* (%)	Kassa et al. [30] *N* (%)	Hailua et al. [31] *N* (%)	Shanko et al. [32] *N* (%)
1	ADR is the same as with side effect	17 (20.73)	30 (23.1)	NA^∗^	65 (30.4)	114 (53.5)	NA^∗^	76 (66.67)	323 (85.2)	82 (35)	127 (41.4)	15 (26.3)	NA^∗^	99 (33.6)
2	Know the term pharmacovigilance	16 (19.51)	NA^∗^	NA^∗^	46 (21.5)	66 (31.0)	NA^∗^	23 (20.18)	176 (46.4)	NA^∗^	90 (29.3)	21 (36.8)	NA^∗^	87 (29.5)
3	Know national ADR reporting system	19 (23.17)	76 (58.5)	284 (69.8)	70 (32.7)	78 (36.6)	NA^∗^	24 (21.05)	222 (58.6)	108 (46.2)	121 (39.4)	34 (59.7)	NA^∗^	175 (59.3)
4	Know the availability of ADR reporting form	21 (25.61)	62 (47.7)	47 (40.2)	NA^∗^	59 (27.7)	31 (37.4)	26 (22.81)	46 (12.1)	134 (68.8)	NA^∗^	36 (63.2)	NA^∗^	181 (61.36)
5	ADR are well documented at the time a drug is marketed	20 (24.39)	25 (19.2)	NA^∗^	NA^∗^	140 (65.7)	NA^∗^	NA^∗^	220 (58)	134 (57.3)	99 (32.2)	NA^∗^	NA^∗^	452 (51.53)
6	Adequately trained in ADR reporting	NA^∗^	22 (16.9)	117 (28.7)	NA^∗^	NA^∗^	NA^∗^	NA^∗^	NA^∗^	NA^∗^	NA^∗^	NA^∗^	NA^∗^	NA^∗^
7	Know responsible body	NA^∗^	NA^∗^	51 (43.6)	22 (10.3)	29 (13.6)	41 (30.8)	35 (30.7)	NA^∗^	92 (39.3)	98 (31.9)	37 (64.9)	98 (65.3)	NA^∗^

^∗^NA: not assessed.

**Table 4:** Attitude of healthcare professionals towards ADR reporting (*n* = 11).

S.no	Questions	Type	Angamo et al. [22] *N* (%)	Bule et al. [23] *N* (%)	Nadew et al. [24] *N* (%)	Kefale et al. [26] *N* (%)	Kasa et al. [28] *N* (%)	Goshime et al. [29] *N* (%)	Denekew et al. [30] *N* (%)	Gidey et al. [31] *N* (%)	Kassa et al. [32] *N* (%)	Hailua et al. [33] *N* (%)	Shanko et al. [34] *N* (%)
1	ADR should be reported spontaneously at regular base	Agree	44 (53.66)	122 (93.8)	NA^∗^	186 (87.3)	88 (77.20)	365 (96.2)	208 (88.9)	207 (67.4)	40 (70.1)	NA^∗^	218 (73.9)
		Disagree	38 (46.44)	8 (6.2)	NA^∗^	27 (12.7)	26 (22.80)	14 (3.8)	26 (11.1)	100 (32.6)	17 (29.9)	NA^∗^	77 (26.1)
2	Reporting ADR is part of duty of health professionals	Agree	47 (57.31)	111 (85.4)	388 (95.3)	179 (84)	100 (87.72)	371 (97.9)	217 (92.7)	NA^∗^	53 (93.0)	NA^∗^	179 (60.68)
		Disagree	35 (42.69)	19 (14.6)	19 (4.7)	34 (16)	14 (22.28)	8 (2.1)	17 (7.3)	NA^∗^	4 (7)	NA^∗^	116 (39.32)
3	Reporting drug safety is important for the public	Agree	59 (71.95)	122 (93.8)	NA^∗^	182 (85.5)	108 (94.74)	366 (96.6)	219 (93.6)	NA^∗^	52 (91.2)	145 (96.7)	246 (83.4)
		Disagree	23 (28.05)	8 (6.2)	NA^∗^	31 (14.5)	6 (5.26)	13 (3.4)	15 (6.4)	NA^∗^	5 (8.8)	5 (3.7)	49 (16.6)
4	Reporting drug safety is important for the health care system	Agree	58 (70.73)	123 (94.6)	258 (63.4)	197 (92.5)	NA^∗^	376 (99.2)	222 (94.9)	135 (44.0)	NA^∗^	NA^∗^	216 (73.2)
		Disagree	24 (29.27)	7 (5.4)	149 (36.6)	15 (7.5)	NA^∗^	3 (0.8)	12 (5.1)	172 (56.0)	NA^∗^	NA^∗^	78 (26.4)
5	There is a need to be sure that ADR is related to the drug before reporting	Agree	70 (85.37)	76 (58.5)	381 (93.6)	164 (77)	87 (76.32)	321 (90)	180 (76.9)	194 (63.2)	NA^∗^	NA^∗^	200 (67.8)
		Disagree	12 (14.63)	54 (41.5)	26 (6.4)	49 (23)	27 (23.68)	38 (10)	54 (23.1)	113 (36.8)	NA^∗^	NA^∗^	95 (32.2)
6	Only ADR of prescription drugs need to be reported	Agree	17 (20.73)	NA^∗^	NA^∗^	NA^∗^	NA^∗^	78 (20.6)	87 (37.2)	82 (26.7)	NA^∗^	NA^∗^	NA^∗^
		Disagree	65 (79.27)	NA^∗^	NA^∗^	NA^∗^	NA^∗^	301 (79.4)	147 (62.8)	225 (73.3)	NA^∗^	NA^∗^	NA^∗^
7	Only ADR that cause persistent disability should be reported	Agree	8 (9.76)	11 (8.5)	NA^∗^	75 (35.2)	48 (42.11)	49 (12.9)	45 (19.2)	NA^∗^	20 (35.0)	NA^∗^	53 (17.97)
		Disagree	74 (90.24)	119 (91.5)	NA^∗^	138 (64.8)	66 (57.89)	330 (87.1)	189 (80.8)	NA^∗^	37 (65)	NA^∗^	242 (82.03)
8	Reporting ADR improves quality of patient care	Agree	60 (73.17)	NA^∗^	391 (96)	NA^∗^	101 (88.60)	NA^∗^	19 (8.1)	148 (48.2)	52 (91.2)	NA^∗^	NA^∗^
		Disagree	22 (26.83)	NA^∗^	16 (4)	NA^∗^	13 (11.40)	NA^∗^	215 (91.9)	159 (51.8)	5 (8.8)	NA^∗^	NA^∗^
9	One report of ADR makes no difference	Agree	14 (17.07)	NA^∗^	45 (11)	NA^∗^	30 (22.32)	NA^∗^	46 (19.7)	103 (33.6)	38 (66.7)	123 (82)	NA^∗^
		Disagree	68 (82.93)	NA^∗^	362 (89)	NA^∗^	84 (73.68)	NA^∗^	178 (80.3)	204 (66.4)	19 (33.3)	27 (18)	NA^∗^
10	Reporting is not useful to the patient	Agree	7 (8.53)	NA^∗^	NA^∗^	21 (10.1)	NA^∗^	14 (3.7)	19 (8.1)	NA^∗^	NA^∗^	NA^∗^	NA^∗^
		Disagree	75 (91.47)	NA^∗^	NA^∗^	192 (89.9)	NA^∗^	365 (96.3)	215 (91.9)	NA^∗^	NA^∗^	NA^∗^	NA^∗^
11	Reporting creates additional work load	Agree	42 (51.21)	46 (35.4)	55 (13.5)	NA^∗^	48 (42.11)	NA^∗^	52 (22.2)	199 (64.8)	NA^∗^	NA^∗^	42 (14.2)
		Disagree	40 (48.79)	84 (64.6)	352 (86.5)	NA^∗^	66 (57.89)	NA^∗^	182 (87.8)	108 (35.2)	NA^∗^	NA^∗^	253 (85.8)
12	Reporting of ADR affects patient's confidentiality issues	Agree	NA^∗^	NA^∗^	35 (8.6)	NA^∗^	31 (27.19)	NA^∗^	NA^∗^	NA^∗^	22 (38.6)	NA^∗^	NA^∗^
		Disagree	NA^∗^	NA^∗^	372 (91.4)	NA^∗^	83 (72.81)	NA^∗^	NA^∗^	NA^∗^	35 (61.4)	NA^∗^	NA^∗^
13	Fear of legal liability affects ADR reporting	Agree	NA^∗^	NA^∗^	209 (51.4)	NA^∗^	60 (52.63)	NA^∗^	NA^∗^	NA^∗^	21 (36.8)	NA^∗^	NA^∗^
		Disagree	NA^∗^	NA^∗^	198 (48.6)	NA^∗^	54 (47.37)	NA^∗^	NA^∗^	NA^∗^	36 (63.2)	NA^∗^	NA^∗^
14	Reporting of ADR should be voluntary	Agree	NA^∗^	NA^∗^	NA^∗^	67 (31.4)	27 (23.68)	200 (52.8)	37 (15.8)	191 (62.2)	17 (29.9)	27 (18)	NA^∗^
		Disagree	NA^∗^	NA^∗^	NA^∗^	146 (68.6)	87 (76.32)	179 (41.2)	197 (84.2)	116 (37.8)	40 (70.1)	123 (82)	NA^∗^

^∗^NA: not assessed.

**Table 5:** Practice of ADR reporting by healthcare professionals (*n* = 13).

S.no	Variable	Type	Angamo et al. [22] *N* (%)	Bule et al. [23] *N* (%)	Nadew et al. [24] *N* (%)	Adimasu et al. [25] *N* (%)	Kefale et al. [26] *N* (%)	Gurmesa et al. [27] *N* (%)	Kasa et al. [28] *N* (%)	Goshime et al. [29] *N* (%)	Denekew et al. [30] *N* (%)	Gidey et al. [31] *N* (%)	Kassa et al. [32] *N* (%)	Hailua et al. [33] *N* (%)	Shanko et al. [34] *N* (%)
1	Have you ever encountered patient with ADR in your clinical practice, in the last 12 months?	Yes	13 (15.85)	84 (66.4)	343 (84.3)	NA^∗^	82 (38.5)	36 (27)	34 (29.82)	173 (45.6)	101 (43.2)	230 (74.9)	12 (21.1)	101 (67.3)	145 (49.2)
		No	69 (84.15)	46 (35.4)	64 (15.7)	NA^∗^	131 (61.5)	97 (73)	80 (70.18)	248 (54.4)	133 (56.8)	77 (25.1)	45 (78.9)	49 (32.6)	149 (50.8)
2	Have you ever reported the ADR?	Yes	0 (0)	38 (29.2)	94 (27.4)	NA^∗^	74 (90.2)	14 (38.8)	17 (50)	28 (16.2)	106 (45.3)	74 (32.1)	10 (83.3)	43 (28.6)	179 (60.68)
		No	82 (100)	92 (70.8)	249 (72.6)	NA ^∗^	8 (9.8)	22 (61.2)	17 (50)	145 (83.8)	128 (54.7)	156 (67.9)	2 (16.7)	107 (71.3)	115 (38.98)
3	How many patients with ADR, did you see?	Zero	NA^∗^	46 (35.4)	NA^∗^	NA^∗^	NA^∗^	NA^∗^	NA^∗^	NA^∗^	NA^∗^	77 (25.1)	NA^∗^	NA^∗^	NA ^∗^
		One	8 (61.54)	22 (16.9)	23 (24.5)	NA^∗^	30 (36.6)	11 (78.5)	11 (9.65)	5 (2.9)	NA^∗^	13 (4.2)	NA^∗^	NA^∗^	30 (10.17)
		Two	3 (23.08)	18 (13.8)	27 (28.7)	NA^∗^	30 (36.6)		8 (7.02)	13 (7.5)	NA^∗^	58 (18.9)	NA^∗^	NA^∗^	41 (13.9)
		Three	1 (7.69)	20 (15.4)		NA^∗^	8 (9.8)		3 (2.63)	25 (14.5)	NA^∗^	61 (19.9)	NA^∗^	NA^∗^	34 (11.53)
		Four	1 (7.69)	6 (4.6)	44(46.8)	NA^∗^	9 (11.0)	3 (21.4)	6 (5.26)	24 (13.9)	NA^∗^	52 (16.9)	NA^∗^	NA^∗^	20 (6.78)
		Above	NA^∗^	18 (13.8)		NA^∗^	5 (6.0)		6 (5.26)	106 (61.3)	NA^∗^	46 (15)	NA^∗^	NA^∗^	21 (7.12)
4	Have you noted the ADR you encountered on the patient clinical record?	Yes	0 (0)	59 (45.4)	299 (87.2)	NA^∗^	NA^∗^	NA^∗^	24 (70.59)	NA^∗^	31 (13.24)	67 (29.1)	10 (83.3)	NA^∗^	110 (37.3)
		No	82 (100)	71 (54.6)	44 (12.8)	NA^∗^	NA^∗^	NA^∗^	10 (29.41)	NA^∗^	75 (32.05)	163 (70.9)	2 (16.7)	NA^∗^	36 (12.2)
5	What kind of ADR need to be reported?	Known reaction	NA^∗^	NA^∗^	NA^∗^	15 (7)	NA^∗^	20 (15)	24 (21.05)	NA^∗^	NA^∗^	NA^∗^	24 (42.1)	25 (16.6)	NA^∗^
		Unknown reaction	NA^∗^	NA^∗^	NA^∗^	35 (16.4)	NA^∗^	21 (15.7)	52 (45.61)	NA^∗^	NA^∗^	NA^∗^	48 (84.2)	24 (16)	NA^∗^
		Life-threatening reaction	NA^∗^	NA^∗^	NA^∗^	149 (69.6)	NA^∗^	33 (24.8)	93 (81.58)	NA^∗^	NA^∗^	NA^∗^	46 (80.7)	81 (54)	NA^∗^
		Do not now	NA^∗^	NA^∗^	NA^∗^	15 (7)	NA^∗^	19 (13.2)	NA^∗^	NA^∗^	NA^∗^	NA^∗^	NA^∗^	20 (13.3)	NA^∗^
6	How often do you give advice to your patients on possible adverse effects of drugs you prescribed, dispensed or administered?	Always	NA^∗^	NA^∗^	NA^∗^	NA^∗^	NA^∗^	NA^∗^	48 (42.11)	NA^∗^	70 (29.9)	31 (10.1)	NA^∗^	NA^∗^	NA^∗^
		Usually	20 (24.39)	43 (33.1)	NA^∗^	NA^∗^	82 (38.5)	NA^∗^	22 (19.3)	45 (11.9)	82 (35)	118 (38.4)	NA^∗^	NA^∗^	101 (34.24)
		Sometimes	35 (42.68)	79(60.8)	NA^∗^	NA^∗^	61 (28.6)	NA^∗^	38 (33.33)	278 (73.4)	17 (7.3)	69 (22.5)	NA^∗^	NA^∗^	180 (61.02)
		Rarely	NA^∗^	NA^∗^	NA^∗^	NA^∗^	53 (24.9)	NA^∗^	6 (5.26)	50 (13.2)	NA^∗^	NA^∗^	NA^∗^	NA^∗^	NA^∗^
		Never	27 (32.93)	8 (6.2)	NA^∗^	NA^∗^	17 (8)	NA^∗^	NA^∗^	6 (1.6)	65 (27.8)	89 (29)	NA^∗^	NA^∗^	12 (4.07)
7	Where did you report the reaction?	Hospital	NA^∗^	8 (6.2)	NA^∗^	NA^∗^	NA^∗^	NA^∗^	8 (47.06)	NA^∗^	NA^∗^	NA^∗^	2 (20)	NA^∗^	102 (34.58)
		Pharmaceutical company	NA^∗^	2 (1.5)	NA^∗^	NA^∗^	NA^∗^	NA^∗^	1 (5.88)	NA^∗^	NA^∗^	NA^∗^	NA^∗^	NA^∗^	41 (13.9)
		EFMHACA	NA^∗^	11 (8.5)	NA^∗^	NA^∗^	8 (10.8)	2 (14.3)	5 (29.41)	0 (0)	NA	NA	4 (40)	NA^∗^	2
		Doctor	NA^∗^	21 (16.2)	NA^∗^	NA^∗^	10 (13.5)	NA^∗^	NA^∗^	NA^∗^	NA^∗^	NA^∗^	NA^∗^	NA^∗^	39 (13.22)
		Never reported	NA^∗^	88 (67.7)	NA^∗^	NA^∗^	NA^∗^	NA^∗^	NA^∗^	NA^∗^	NA^∗^	NA^∗^	NA^∗^	NA^∗^	NA^∗^
		Head pharmacy	NA^∗^	NA^∗^	NA^∗^	NA^∗^	28 (37.8)	NA^∗^	8 (47.06)	17 (60.7)	NA^∗^	NA^∗^	4 (40)	NA^∗^	NA^∗^
		Manufacturers	NA^∗^	NA^∗^	NA^∗^	NA^∗^	6 (8.1)	NA^∗^	NA^∗^	10 (35.7)	NA^∗^	NA^∗^	0 (0)	NA^∗^	NA^∗^
		Ministry of health	NA^∗^	NA^∗^	NA^∗^	NA^∗^	16 (21.6)	NA^∗^	2 (11.76)	NA^∗^	NA^∗^	NA^∗^	0 (0)	NA^∗^	NA^∗^
		Others^∗^	NA^∗^	NA^∗^	NA^∗^	NA^∗^	6 (8.2)	12 (85.7)	NA^∗^	1 (3.6)	NA^∗^	NA^∗^	NA^∗^	NA^∗^	NA^∗^
8	How are ADR reported?	Telephone	NA^∗^	14 (10.8)	NA^∗^	NA^∗^	NA^∗^	NA^∗^	NA^∗^	NA^∗^	NA^∗^	NA^∗^	NA^∗^	37 (24.6)	182 (61.69)
		Post	NA^∗^	56 (43.1)	NA^∗^	NA^∗^	NA^∗^	NA^∗^	NA^∗^	NA^∗^	NA^∗^	NA^∗^	NA^∗^	48 (32)	
		E-mail	NA^∗^	18 (13.8)	NA^∗^	NA^∗^	NA^∗^	NA^∗^	NA^∗^	NA^∗^	NA^∗^	NA^∗^	NA^∗^	11 (7.3)	
		Do not know	NA^∗^	42 (32.3)	NA^∗^	NA^∗^	NA^∗^	NA^∗^	NA^∗^	NA^∗^	NA^∗^	NA^∗^	NA^∗^	54 (36)	113 (38.64)

^∗^Others: Ethiopian pharmaceutical association, wholesales, nurse head, drug therapeutic committee; NA: not assessed.

**Table 6:** Health care professionals perceived factors for ADR underreporting (*n* = 4).

S.no	Perceived factors	Kasa et al. [28] *N* (%)	Goshime et al. [29] *N* (%)	Denekew et al. [30] *N* (%)	Shanko et al. [34] *N* (%)
1	Concern that the report may be wrong	87 (76.3)	NA^∗^	100 (42.7)	NA^∗^
2	Not knowing how to fill and report ADR	57 (50)	NA^∗^	NA^∗^	NA^∗^
3	Uncertain of causal association between drug and ADR	61 (53.51)	310 (81.8)	NA^∗^	153 (51.9)
4	Lack of time to fill report form	78 (68.42)	NA^∗^	67 (28.6)	64 (21.7)
5	Reporting does not influence the treatment scheme	76 (66.67)	NA^∗^	NA^∗^	NA^∗^
6	Forgetfulness	56 (49.12)	NA^∗^	NA^∗^	40 (13.6)
7	Lack of feedback	33 (28.95)	NA^∗^	NA^∗^	121 (41)
8	Fear of legal liability by reporting ADR	59 (51.75)	117 (30.9)	56 (23.9)	NA^∗^
9	Concern that a report will generate an extra work	71 (62.28)	101 (26.6)	61 (26.1)	NA^∗^
10	Belief that only safe drugs are marketed	80 (70.17)	NA^∗^	80 (34.2)	NA^∗^
11	Thinking that one report does not make any difference	75 (65.78)	49 (12.9)	57 (24.4)	NA^∗^
12	Thinking that you may have caused a patient harm	75 (69.3)	NA^∗^	NA^∗^	NA^∗^
13	My report is not needed/necessary	90 (78.94)	15 (4.0)	39 (16.7)	NA^∗^
14	Insufficient clinical knowledge	57 (50)	NA^∗^	99 (42.3)	NA^∗^
15	Reporting forms are not available when needed	45 (39.48)	341 (90.0)	95 (40.6)	159 (53.9)
16	Thinking that ADR reporting is not a duty	84 (73.69)	NA^∗^	NA^∗^	NA^∗^
17	Not knowing where to report	48 (42.11)	NA^∗^	NA^∗^	NA^∗^
18	Other colleagues are not reporting ADR cases	49 (42.99)	NA^∗^	84 (35.9%)	NA^∗^
19	No ADR reporting system	NA^∗^	160 (42.2)	84 (35.9)	NA^∗^
20	Lack of motivation	NA^∗^	190 (50.3)	NA^∗^	NA^∗^

^∗^NA: not assessed.

## Acronyms

ADR: Adverse drug reactions
EFDA: Ethiopian Food and Drug Administration.

## References

[1] Hanafi S., Torkamandi H., Hayatshahi A., Gholami K., Javadi M. (2012). Knowledge, attitudes and practice of nurse regarding adverse drug reaction reporting. *Iranian Journal of Nursing and Midwifery Research*.

[2] World Health Organization (2004). *WHO Guide Lines on Safety Monitoring of Herbal Medicines in Pharmacovigilance Systems*.

[3] Organization WH (2002). *The Selection of Essential Medicines*.

[4] Ji Y., Ying H., Dews P. (2011). A potential causal association mining algorithm for screening adverse drug reactions in postmarketing surveillance. *IEEE Transactions on Information Technology in Biomedicine*.

[5] Kamtane R. A., Jayawardhani V. (2012). Knowledge, attitude and perception of physicians towards adverse drug reaction (ADR) reporting: a pharmacoepidemiological study. *Asian Journal of Pharmaceutical and Clinical Research*.

[6] Pouyanne P., Haramburu F., Imbs J. L., Bégaud B. (2000). Admissions to hospital caused by adverse drug reactions: cross sectional incidence study. *BMJ*.

[7] Pharmacovigilance, WHO (2004). *Ensuring the safe use of medicines, WHO policy perspective of medicine*.

[8] Chen Y. C., Fan J. S., Hsu T. F. (2012). Detection of patients presenting with adverse drug events in the emergency department. *Internal Medicine Journal*.

[9] Wester K., Jönsson A. K., Spigset O., Druid H., Hägg S. (2008). Incidence of fatal adverse drug reactions: a population based study. *British Journal of Clinical Pharmacology*.

[10] Bouvy J. C., De Bruin M. L., Koopmanschap M. A. (2015). Epidemiology of adverse drug reactions in Europe: a review of recent observational studies. *Drug Safety*.

[11] Hodgkinson M. R., Dirnbauer N. J., Larmour I. (2009). Identification of adverse drug reactions using the ICD-10 Australian modification clinical coding surveillance. *Journal of Pharmacy Practice and Research*.

[12] Tegegne G. T., Gaddisa T., Kefale B., Tesfaye G., Likisa J. (2015). *Drug Therapy Problem and Contributing Factors among Ambulatory Hypertensive Patients in Ambo General Hospital*.

[13] Oumer S. (2017). Irrational use of medications among elderly patients in an Ethiopian referral hospital. *African Journal of Pharmacy and Pharmacology*.

[14] Hunchak C., Teklu S., Meshkat N., Meaney C., Puchalski Ritchie L. (2015). Patterns and predictors of early mortality among emergency department patients in Addis Ababa, Ethiopia. *BMC Research Notes*.

[15] Palaian S., Ibrahim M. I., Mishra P. (2011). Health professionals' knowledge, attitude and practices towards pharmacovigilance in Nepal. *Pharmacy practice.*.

[16] FMHACA (2014). *Guideline for Adverse Drug Events Monitoring (Pharmacovigilance)*.

[17] EFDA (2014). *Guidelines for Medicine Registration in: 3rd Edition*.

[18] Zolezzi M., Parsotam N. (2005). Adverse drug reaction reporting in New Zealand: implications for pharmacists. *THERAPEUTICS and Clinical Risk Management*.

[19] Gupta P., Udupa A. (2011). Adverse drug reaction reporting and pharmacovigilance: knowledge, attitudes and perceptions amongst resident doctors. *Journal of Pharmaceutical Sciences and Research*.

[20] Fadare J. O., Enwere O. O., Afolabi A., Chedi B., Musa A. (2011). Knowledge, attitude and practice of adverse drug reaction reporting among healthcare workers in a tertiary centre in Northern Nigeria. *Tropical Journal of Pharmaceutical Research*.

[21] Dissemination C. (2009). *CRD’s Guidance for Undertaking Reviews in Health Care*.

[22] Angamo M. T., Tesfa A., Wabe N. T. (2012). Knowledge, attitude and practice of adverse drug reaction reporting among health professionals in Southwest Ethiopia. *TAF Preventive Medicine Bulletin*.

[23] Bule M. H., Hamido B. A., Chala T. S., Kefeni G. T. (2016). Knowledge, attitudes and practices of healthcare professionals towards adverse drug reaction reporting in Adama hospital medical college, east Shoa zone, Oromia regional state, Ethiopia. *The Pharma Innovation*.

[24] Nadew S. S., Beyene K. G./. M., Beza S. W. (2020). Adverse drug reaction reporting practice and associated factors among medical doctors in government hospitals in Addis Ababa, Ethiopia. *Plos One*.

[25] Adimasu A. (2014). Nurses knowledge related to adverse drug reaction reporting and associated factors at Felegehiwot Referral Hospital and University of Gondar Teaching Hospital, Northwest Ethiopia. *American Journal of Health Research*.

[26] Kefale A. T., Tefera B. D., Biru T. T. (2017). Knowledge, attitude and practice of healthcare professionals towards adverse drug reaction reporting at inpatient wards of tertiary hospital, Ethiopia. *Journal of Drug Delivery and Therapeutics*.

[27] Gurmesa L. T., Dedefo M. G. (2016). Factors affecting adverse drug reaction reporting of healthcare professionals and their knowledge, attitude, and practice towards ADR reporting in Nekemte Town, West Ethiopia. *Bio Med research international*.

[28] Kassa Alemu B., Biru T. T. (2019). Health care professionals’ knowledge, attitude, and practice towards adverse drug reaction reporting and associated factors at selected public hospitals in Northeast Ethiopia: a cross-sectional study. *Bio Med Research International*.

[29] Goshime A. (2015). *Assessment of Knowledge, Attitude and Practices on Adverse Drug Reaction Reporting among Pharmacy Personnel Working at Community Pharmacy*.

[30] Denekew A. (2014). *Knowledge, Attitude and Practice of Adverse Drug Reaction Reporting and Affecting Factors among Health Care Providers Working in Art Clinics of Public Health Facilities in Addis Ababa City*.

[31] Gidey K., Seifu M., Hailu B. Y., Asgedom S. W., Niriayo Y. L. (2020). Healthcare professionals knowledge, attitude and practice of adverse drug reactions reporting in Ethiopia: a cross-sectional study. *BMJ Open*.

[32] Kassa B., Mulu A., Geresu B. (2017). Health care providers knowledge, attitude and experience of adverse drug reaction reporting. *African Journal of Pharmacy and Pharmacology*.

[33] Hailua W., Bhagavathulab A. S., Admassiec E., Pateld I., Khane T. M. (2014). Knowledge, attitude and practices towards adverse drug reaction reporting in Gondar, Ethiopia. *Age*.

[34] Shanko H., Abdela J. (2018). Knowledge, attitudes, and practices of health care professionals toward adverse drug reaction reporting in Hiwot Fana Specialized University Hospital, Harar, Eastern Ethiopia: a cross-sectional study. *Hospital Pharmacy*.

[35] Chyka P. A. (2000). How many deaths occur annually from adverse drug reactions in the United States?. *The American journal of medicine*.

[36] Lundkvist J., Jönsson B. (2004). Pharmacoeconomics of adverse drug reactions. *Fundamental & clinical pharmacology*.

[37] JFDA (2014). *Guidelines for Detecting & Reporting Adverse Drug Reactions: Individual Case Safety Reports for Healthcare Professionals, Rational Drug Use and Pharmacovigilance Department*.

[38] Vallano A., Cereza G., Pedròs C. (2005). Obstacles and solutions for spontaneous reporting of adverse drug reactions in the hospital. *British journal of clinical pharmacology.*.

[39] Bhagavathula A. S., Elnour A. A., Jamshed S. Q., Shehab A. (2016). Health professionals’ knowledge, attitudes and practices about pharmacovigilance in India: A systematic review and meta-analysis. *PloS one*.

[40] Abubakar A. R., Simbak N. B., Haque M. (2014). A systematic review of knowledge, attitude and practice on adverse drug reactions and pharmacovigilance among doctors. *Journal of Applied Pharmaceutical Science*.

[41] Bailey C., Peddie D., Wickham M. E. (2016). Adverse drug event reporting systems: a systematic review. *British journal of clinical pharmacology*.

[42] World Health Organization (2002). *The Importance of Pharmacovigilance*.

[43] Hazell L., Shakir S. A. (2006). Under-reporting of adverse drug reactions. *Drug Safety*.

[44] Al Dweik R., Stacey D., Kohen D., Yaya S. (2017). Factors affecting patient reporting of adverse drug reactions: a systematic review. *British journal of clinical pharmacology*.

[45] Varallo F. R., Guimarães S. O. P., Abjaude S. A. R., Mastroianni P. C. (2014). Causes for the underreporting of adverse drug events by health professionals: a systematic review. *Revista da Escola de Enfermagem da USP*.

[46] Molokhia M., Tanna S., Bell D. (2009). Improving reporting of adverse drug reactions: systematic review. *Clinical Epidemiology*.

[47] Ribeiro-Vaz I., Silva A. M., Costa Santos C., Cruz-Correia R. (2016). How to promote adverse drug reaction reports using information systems–a systematic review and meta-analysis. *BMC Medical Informatics and Decision Making*.

